# Mapping Expression Quantitative Trait Loci Targeting Candidate Genes for Pregnancy in Beef Cows

**DOI:** 10.3390/biom14020150

**Published:** 2024-01-26

**Authors:** Wellison J. S. Diniz, Juliana Afonso, Nicholas C. Kertz, Paul W. Dyce, Priyanka Banerjee

**Affiliations:** 1 Department of Animal Sciences, Auburn University, Auburn, AL 36849, USA; nck0012@auburn.edu (N.C.K.); pwd0003@auburn.edu (P.W.D.); pzb0035@auburn.edu (P.B.); 2 Embrapa Pecuária Sudeste, Rodovia Washington Luiz, Km 234, s/n, Fazenda Canchim, São Carlos 13560-970, SP, Brazil; juafonsobio@gmail.com

**Keywords:** cow fertility, eQTL, gene expression, regulatory variants, reproduction

## Abstract

Despite collective efforts to understand the complex regulation of reproductive traits, no causative genes and/or mutations have been reported yet. By integrating genomics and transcriptomics data, potential regulatory mechanisms may be unveiled, providing opportunities to dissect the genetic factors governing fertility. Herein, we identified regulatory variants from RNA-Seq data associated with gene expression regulation in the uterine luminal epithelial cells of beef cows. We identified 4676 cis and 7682 trans eQTLs (expression quantitative trait loci) affecting the expression of 1120 and 2503 genes, respectively (FDR < 0.05). These variants affected the expression of transcription factor coding genes (71 cis and 193 trans eQTLs) and genes previously reported as differentially expressed between pregnant and nonpregnant cows. Functional over-representation analysis highlighted pathways related to metabolism, immune response, and hormone signaling (estrogen and GnRH) affected by eQTL-regulated genes (*p*-value ≤ 0.01). Furthermore, eQTLs were enriched in QTL regions for 13 reproduction-related traits from the CattleQTLdb (FDR ≤ 0.05). Our study provides novel insights into the genetic basis of reproductive processes in cattle. The underlying causal mechanisms modulating the expression of uterine genes warrant further investigation.

## 1. Introduction

Female fertility and reproductive success are the main drivers of economic efficiency in cattle operations. While management practices and the adoption of new reproductive technologies have addressed the declining trend in fertility, its genetic progress has been limited [1]. There is significant genetic variation associated with fertility traits; however, their improvement has been challenging due to their complex and multifactorial nature [2,3]. To address this challenge, efforts from breeding programs worldwide have focused on identifying novel fertility phenotypes [4,5]. Similarly, the emergence of next-generation sequencing technologies and genomic selection have opened new avenues to increase reproductive efficiency [2].

Genome-wide association studies have been extensively used to dissect the complex genetic basis of cow fertility [6,7,8]. Although promising candidate genes have been reported, most variants identified have a small effect size [6] and are located in noncoding regions [9,10]. Recently, new approaches have focused on functional information from reproductive-related tissues [11]. Ross et al. [12] reported extensive gene expression variation in candidate genes across 11 tissues in Brahman cattle. Similarly, changes in the expression profile of uterine tissue were associated with differences in fertility status and reproductive success in cows [8,13,14]. Such changes are related to the morphological and functional programming of the endometrium to support the attachment of the conceptus [14,15]. Signaling pathways involved with tissue remodeling, the immune system, and inflammatory response were shared across studies investigating fertility [14,15,16]. Yet, there is limited overlapping of candidate genes among them [17].

Considering the complex regulatory mechanisms underlying fertility, further advances will rely on holistic approaches to identify key genomic factors and their causal relationships [17,18]. Among these approaches, expression quantitative trait loci (eQTL) effectively integrate genetic variants controlling gene expression genome-wide. Expression QTL studies have been reported for several complex traits; however, only a few of them have focused on fertility in cattle. Kadarmideen and Mazzoni [19] reported eQTLs affecting genes in the bovine follicular cells and the endometrium of dairy cows. The eQTL-modulated genes were associated with the quality of in vitro-produced embryos and endometrial receptivity [19]. Berg et al. [6] reported eQTLs underlying the expression of genes from white blood cells associated with the calving interval in dairy cows. Forutan et al. [10] identified putatively functional genes affecting cattle fertility by integrating genome-wide association results and eQTLs for whole blood from female Brahman cattle. Despite these findings, we still have a limited understanding of the single-nucleotide polymorphisms (SNPs) regulating the genes involved with fertility-related traits.

Here, we used a genomics–transcriptomics approach to identify the regulatory variants associated with pregnancy status and gene expression regulation in beef cows. We hypothesized that genetic variants modulate the gene expression of uterine luminal epithelial cells, affecting the biological processes and pathways underlying reproduction. To test our hypothesis, we first identified SNPs affecting the expression of genes from the uterine luminal epithelial cells. Next, we investigated if the SNPs were associated with pregnancy outcomes, affecting differentially expressed genes, and/or harbored in known QTL regions for reproduction traits. Lastly, we identified enriched pathways and biological processes that underlie the eQTL-modulated genes.

## 2. Materials and Methods

**Figure 1:**
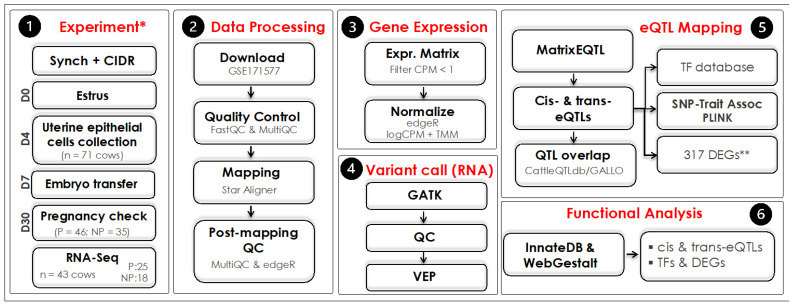
Experimental design and data integration outline to dissect the eQTLs from the uterine luminal epithelial cells of beef cows. * Experimental project and ** differentially expressed genes were performed, and previously published by Martins et al. [14]. P: pregnant; NP: nonpregnant; QC: quality control; QTL: quantitative trait loci.

We used RNA-Seq data from the GEO database (accession number GSE171577, BioProject PRJNA720121). The experiment was performed, and results were published by Martins et al. [14]. A summary of the approach used in the current study is shown in Figure 1.

Martins et al. [14] investigated the uterine luminal epithelial profile of 43 multiparous Angus–Brahman cows. Briefly, uterine luminal epithelial cells were collected from estrous-synchronized recipient cows three days before embryo transfer (ET) using a cytological brush. The average body weight and age of the recipient cows were 556.8 ± 9 kg and 6.7 ± 0.3 years, respectively. Cows were kept grazing and supplemented with hay, concentrate, and minerals. Pregnancy was diagnosed on day 30 after ET (25 pregnant—P; and 18 nonpregnant—NP) through transrectal ultrasonography. Further details on the experimental design and procedures were described elsewhere [14].

### 2.1. Data Collection, Quality Control, and RNA-Seq Mapping

The data were downloaded from the GEO database using the SRA-Explorer web tool v.1.0 [20] and processed using a custom-built bioinformatics pipeline, as previously described [21]. First, FastQC v0.11.9 [22] and MultiQC v1.11 [23] software were used for the quality control (QC) and aggregation of results, respectively. Next, we performed a two-pass alignment using the STAR aligner v2.7.5 [24]. The *Bos taurus* genome ARS-UCD 1.2 was used as the reference based on the assembly and annotation files from the Ensembl database (release 104). Additionally, we used the *quantMode GeneCounts* flag from STAR for counting the mapped reads and *outSAMtype BAM SortedByCoordinate* to sort the .bam file. Raw counts were transformed to counts per million (CPM) using the edgeR package v3.40.2 [25]. Then, non- or low-expressed genes (CPM < 0.5 in 50% of the samples) were filtered out. The remaining genes were transformed to log2CPM and normalized following the TMM method (trimmed mean of M values) on edgeR.

### 2.2. RNA-Seq Variant Calling and Quality Control

RNA-Seq variants were identified following the Genome Analysis Toolkit (GATK, v. 4.1.9.0) software and best practices for RNA-seq data [26,27,28]. We used the Ensembl *B. taurus* dbSNP (release 96) as known variants for recalibration. Genetic variants for each sample were identified and recorded in Individual Genomic VCF (GVCF) files using the *HaplotypeCaller* algorithm. The files were then merged, and all samples were jointly genotyped. Only biallelic SNPs were kept for further analysis. We used vcftools v0.1.17 [29] for stringent quality control, following the GATK hard-filtering recommendations: the variant quality score (QUAL) ≥ 30 and total depth of coverage (DP) > 10. Additionally, variants with a call rate < 95%, a minor allele frequency (MAF) < 5%, and located in sexual chromosomes were filtered out. A single VCF file with 43 samples and all genotypes was generated and used for further analysis.

We used a principal component analysis (PCA) based on the variance-standardized relationship matrix from PLINK (v1.90b6.2) to investigate population stratification. Considering that no population stratification was observed, we performed SNP pruning (tag SNP) based on linkage disequilibrium (LD) thresholds as a second quality-assurance step. This procedure removed the SNPs with similar effects, so those remaining SNPs represented the LD block. The parameters used for the LD pruning were as follows: *r*^2^ = 0.8, a window size of 100 SNPs, and a window offset every 10 SNPs at each step.

### 2.3. Variant Functional Prediction

To compute the functional effects of the genomic variants, we used the Ensembl Variant Effect Predictor (VEP) software v.101 [30]. Upstream and downstream annotations for the variants were assigned within 5 kb. Furthermore, the SIFT (sorting intolerant from tolerant) score within the VEP was used to predict the effects of the amino acid changes in the protein function.

### 2.4. The eQTL Mapping and Annotation

To identify the SNPs associated with the expression levels of the genes in the uterine luminal epithelial cells, we performed an integrative analysis using MatrixEQTL v2.1.1 [31]. To this end, we performed a linear association between each SNP (tag SNP) and each gene, assuming an additive effect [31]. We included the block as a covariate in the model, representing the round (n = 2) of the ET trial [14]. We performed two separate tests for each gene–SNP to identify cis (SNPs within 1 Mb upstream or downstream of the gene) and trans eQTLs (SNPs within more than 1 Mb of the associated gene) [31]. The *p*-values were adjusted by computing the false discovery rate (FDR) using the Benjamini–Hochberg procedure [32]. The eQTLs with an FDR < 0.05 were considered significant. The SNPs were mapped to the *B. taurus* genome ARS-UCD1.2, and gene annotations were retrieved from the Ensembl database using BiomaRt v2.54.1 [33].

Expression QTLs can also affect gene expression by regulating transcription factors (TFs). Thus, we investigated if the genes encoding TFs were affected by the eQTLs. To this end, we downloaded 1396 TFs from the Animal Transcription Factor Database v3.0 [34] and overlapped them with the list of cis and trans eQTLs. Additionally, we investigated if the genes and TF-coding genes were differentially expressed in the uterine luminal epithelial cells. Therefore, we retrieved the list of differentially expressed genes (DEGs, n = 317) associated with pregnancy outcomes reported by Martins et al. [14]. Lastly, we searched for regions in the genome over-represented by DEGs. We used the ShinyGO v.077 web tool to scan the *B. taurus* genome (window size = 6 Mb). We performed a hypergeometric test within each window to determine if the number of genes was significantly over-represented (FDR ≤ 0.05).

### 2.5. SNP Trait Association and Cattle QTLdb Over-Representation Analyses

The SNPs identified as eQTLs were retrieved and used for a trait association analysis. Each SNP was associated with the pregnancy status using a logistic regression model on PLINK, including the block (two rounds of ET) as a covariate. We used logistic regression due to the binary nature of the pregnancy outcome (NP or P). This strategy allows us to predict the log odds ratio associated with each additional copy of the minor allele to be associated with the pregnancy status.

To investigate if the eQTLs were overlapping with known quantitative trait loci (QTLs), we used the GALLO R-package v.1.3 [35]. Known QTLs were downloaded from the Animal Genome cattle QTLdb database (release 49) [36]. Next, the eQTLs were annotated using the *find_genes_qtls_around_markers* function from GALLO. Separate analyses were performed for cis and trans eQTLs. The QTL windows were defined as 1 Mb intervals (500 Kb upstream and 500 Kb downstream) [35]. Lastly, we performed a chromosome-based QTL enrichment analysis and retrieved the significant results associated with *QTL_type = = “Reproduction”*. The *p*-values were adjusted to multiple testing corrections based on the FDR, and significant results were taken when the FDR ≤ 0.05.

### 2.6. Functional Over-Representation Analysis

To identify the biological processes and pathways underlying the genes affected by the eQTLs, we performed a functional over-representation analysis (ORA) using the InnateDB v.5.4 [37] and WebGestalt v.0.46 [38] tools. We examined the genes regulated by cis and trans eQTLs separately. Common genes targeted by both cis and trans eQTLs were analyzed as well. The over-representation analysis was performed using a hypergeometric test, and significant results were retrieved considering a *p*-value ≤ 0.01. Furthermore, we analyzed the DEG list and identified pathways harboring eQTL-affected genes.

## 3. Results

Here, we performed an eQTL mapping analysis to identify regulatory variants associated with gene expression regulation in beef cows. We used public RNA-Seq data to characterize the variants (SNPs) from the uterine luminal epithelial cells of cows retrospectively classified as pregnant or nonpregnant. Using this approach, we:

1. Identified the SNPs affecting the expression of genes from the uterine luminal epithelial cells;
2. Investigated if the SNPs were associated with pregnancy outcomes, affected differentially expressed genes, and/or were harbored in known QTL regions for reproduction traits;
3. Identified over-represented pathways and biological processes that underlie eQTL-modulated genes.

### 3.1. Variant Calling Analysis Retrieved 203,404 Unique SNPs

From the 43 samples analyzed here, we retrieved, on average, 22.14 million reads (ranging from 17.3 to 29.8 M per sample) uniquely mapped to the *B. taurus* reference genome (Supplementary Table S1). We filtered out low- or nonexpressed genes and normalized the read counts through CPM and log2 transformation. A total of 15,029 genes were expressed in uterine epithelial cells based on our QC criteria (see Materials and Methods, Section 2.1) and used for further analysis. The RNA-Seq variant calling based on the GATK pipeline resulted in 4,859,784 SNPs. After filtering (see Materials and Methods, Section 2.2), 203,404 unique variants were kept for further analysis.

We used the Ensembl VEP analysis to predict the functional consequences of the detected SNPs. We identified 16,926 (8.3%) novel variants out of the 203,404 SNPs tested, overlapping 15,055 genes on the reference genome. This analysis revealed that most variants were in Chr 19, 18, and 3 (12,297, 11,733, and 11,547 SNPs, respectively). The most severe consequences predicted by the VEP showed that the variants were mainly harbored downstream (44,161 SNPs) or on 3′-untranslated regions (UTR—39,958 SNPs). Furthermore, we identified 37,794 missense variants. We used the SIFT score to predict the effects of the amino acid changes on the protein function. Although most mutations were classified as tolerated (70.6%), we found 12.6% with a deleterious effect. Supplementary Table S2 shows the results from the VEP and SIF analyses. The distribution and consequences of these variants are reported in Supplementary Tables S3 and S4.

### 3.2. Cis and Trans eQTLs Affected the Expression of 3157 Genes

**Figure 2:**
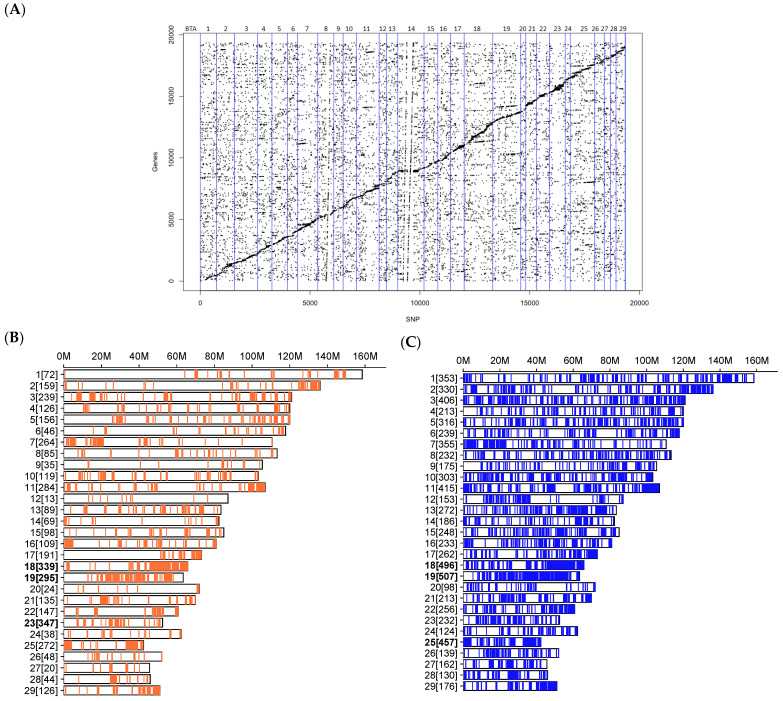
Chromosome distribution of the eQTLs identified from bovine uterine luminal epithelial cells. (**A**) Scatter plot of the gene location vs. eQTL position in Mb. Cis and trans eQTLs are scattered diagonally and vertically, respectively (FDR < 0.05). (**B**,**C**) The number of significant cis and trans eQTLs per chromosome between brackets, respectively. The top three chromosomes are highlighted in bold.

To understand the effects of the variants on gene abundance, we integrated the expression data from uterine luminal epithelial cells to the SNPs detected from the RNA-Seq of 43 cows. To reduce the false discovery rate, we performed SNP pruning based on the LD. The eQTLs for 15,029 genes and 10,879 LD-pruned SNPs (tag SNPs) were identified by a linear model using the MatrixEQTL R-package. Figure 2 shows the distribution of the eQTLs across the genome. We identified 4676 cis eQTLs (SNPs within 1 Mb of the associated gene) corresponding to 3989 unique SNPs affecting the expression of 1120 genes (Supplementary Table S3) (FDR ≤ 0.05). Chromosomes 23, 18, and 19 harbored the most cis-acting SNPs (347, 339, and 295, respectively) (Figure 2B). The top-five targeted genes included *ENSBTAG00000053827*, *ENSBTAG00000016148*, *AIFM3*, *TMEM69*, and *ENSBTAG00000026758*, affected by 93, 65, 61, 51, and 48 SNPs, respectively. Among the cis-regulated genes, we identified 71 coding TF genes modulated by at least one SNP (ranging from 1 to 35 SNPs). Additionally, the TFs from the zf-C2H2 family were redundantly regulated, including *ZNF420*, *ZNF623*, and *ZNF775*, modulated by 35, 22, and 19 cis eQTLs.

Regarding the SNPs within more than 1 Mb of the associated gene or in other chromosomes, we identified 14,680 trans eQTLs corresponding to 7682 unique SNPs affecting the expression of 2503 genes (Supplementary Table S4) (FDR ≤ 0.05). Chromosomes 19, 18, and 25 harbored the most trans-acting SNPs (507, 496, and 457, respectively) (Figure 2C). The top-five targeted genes included *ENSBTAG00000007816*, *PPP1R3D*, *ENSBTAG00000052527*, *ENSBTAG00000027075*, and *ENSBTAG00000048353,* affected by 195, 192, 178, 171, and 151 eQTLs, respectively. The trans eQTLs affected the expression of 193 TFs from 42 families. Like the cis eQTLs, most of the TFs were from the zf-C2H2 family (73 genes), followed by bHLH (10) and ZBTB (10). The top-five TFs included *ZNF420*, *ENSBTAG00000015866*, *HES4*, *ZBTB32*, and *ZKSCAN2*, affected by 128, 116, 67, 42, and 37 eQTLs, respectively. Among the novel variants, 315 were cis and 2425 (708 unique SNPs) were trans eQTLs. Lastly, we identified 4297 eQTLs (corresponding to 792 SNPs) acting as cis and trans eQTLs and affecting 466 genes (Supplementary Table S5). Table 1 shows the top-ten genes and the number of eQTLs. While most of them are protein-coding genes, the *ENSBTAG00000052527* encodes a lncRNA.

### 3.3. eQTLs Were Over-Represented in Reproduction-Related QTL Regions

**Figure 3:**
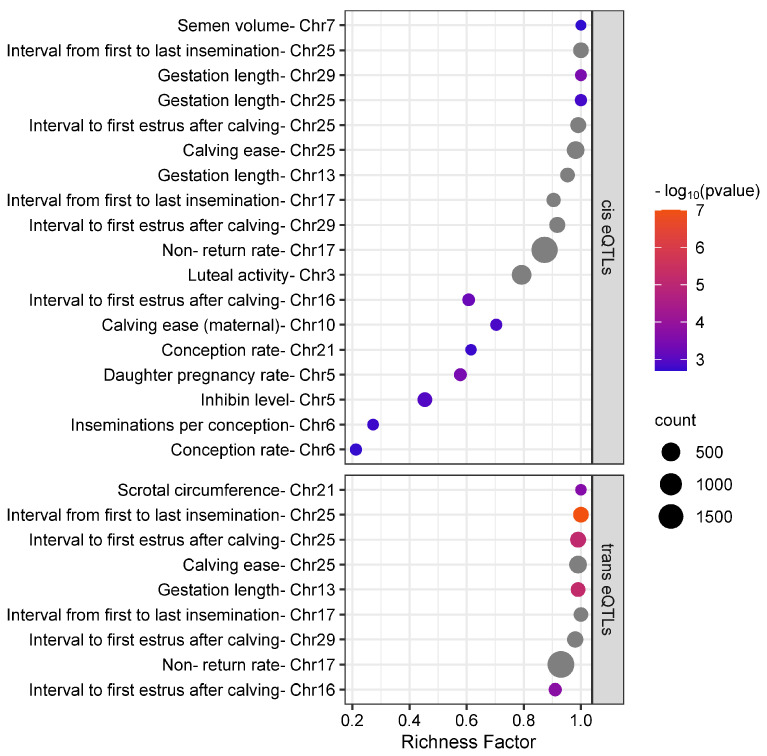
Over-represented reproduction-related traits (QTLs) from cattle QTLdb overlapping cis and trans eQTLs identified from bovine uterine luminal epithelial cells. The *x-axis* represents the ratio between the number of observed QTLs and the expected number of that QTL, while the *y-axis* represents the over-represented traits. The color scale indicates the -log10 (*p*-value) and the dot size indicates the number of observed QTLs for that trait. Only significant traits are shown (FDR ≤ 0.05).

We performed a SNP trait association to investigate the effects of the SNPs on pregnancy outcomes. Based on the association analysis in PLINK, we did not find any significant SNP (FDR > 0.05). We then investigated if the eQTLs identified here overlapped with known QTLs for reproductive-related traits from the Animal Genome cattle QTLdb database. We identified 18 and 9 significantly over-represented QTLs by cis and trans eQTLs, respectively (Figure 3) (FDR ≤ 0.05). The overlapping cis and trans eQTLs were spread across 11 and 6 chromosomes, respectively. The top three over-represented traits overlapped by cis eQTLs included the nonreturn rate, luteal activity, and gestation length. From the overlapping trans eQTLs, in addition to the nonreturn rate, the top traits include the interval to the first estrus after calving and the interval from the first to last insemination.

### 3.4. Differentially Expressed Genes between Pregnant and Nonpregnant Cows Were Affected by eQTLs

To focus on the SNPs with potential functional roles in reproduction, we overlapped the 317 DEGs affecting pregnancy outcomes from Martins et al. [14] with the genes reported here. We identified 32 and 56 DEGs affected by cis and trans eQTLs (Supplementary Table S6). Among them, the *ZNF470* and *SNAPC4* TFs were affected by cis eQTLs, while *PPARG*, *AR*, and *KLF7* were affected by trans eQTLs. Additionally, 13 DEGs were affected by both cis and trans eQTLs. Interestingly, we found 13 regions over-represented by DEGs on chromosomes 18, 19, 22, and 25 (FDR ≤ 0.05) (Table 2).

### 3.5. eQTL-Modulated Genes Are Over-Represented in Pathways Related to the Metabolism and Immune Response

We performed a functional over-representation analysis to understand the biological processes and pathways affected by the genes targeted by eQTLs. The analysis of cis-targeted genes retrieved eight significant pathways, including the tryptophan metabolism, phenylalanine and tyrosine catabolism, and metabolism of lipids and lipoproteins (*p* ≤ 0.01). Immune-related biological processes affected by those genes included antigen processing and the presentation of peptide antigen via MHC class I and immune response (Supplementary Table S7). Similarly, the FOXM1 transcription factor network, ubiquitin-mediated proteolysis, and coregulation of androgen receptor activity were among the over-represented pathways by trans eQTL-affected genes. We also identified 50 significant over-represented biological processes (BPs) (Supplementary Table S8), including the MAPK cascade, embryonic placenta development, and the response to estrogen.

**Figure 4:**
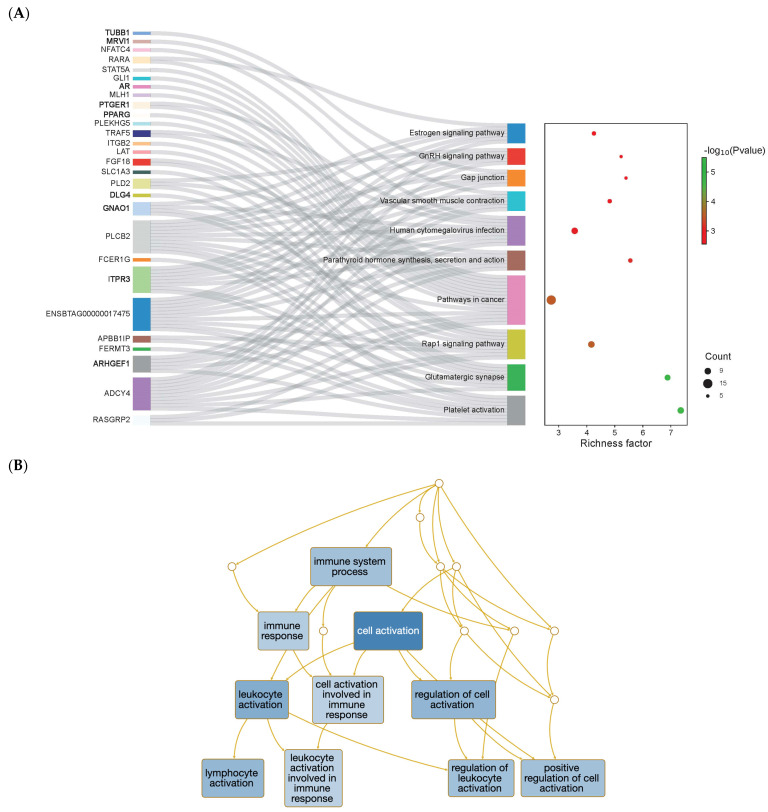
Over-represented functional categories for differentially expressed genes (DEGs) from bovine uterine luminal epithelial cells. DEGs were retrieved from Martins et al. [14]. Functional analysis results were based on WebGestalt. (**A**) Sankey and dot plots showing the pathways connected to the DEGs. Genes affected by eQTLs are highlighted in bold. The color scale indicates the −log10 (*p*-value), and the size of the dot indicates the number of observed genes for that pathway. (**B**) Directed acyclic graph of the significant biological processes. The darker the blue background color in the squares, the more significant is the term (*p*-value ≤ 0.01).

Genes affected by both cis and trans eQTLs were involved in pathways such as the metabolism of amino acids and derivatives, fatty acid degradation, and the tryptophan metabolism (*p* ≤ 0.01). Furthermore, antigen processing and presentation, the immune response, and the positive regulation of interferon-alpha production were among the over-represented BPs (Supplementary Table S9). Over-represented BPs and pathways affected by eQTLs were underlying gene expression regulation. The gene ontology (GO) terms included the regulation of transcription DNA-templated and the positive regulation of transcription. Likewise, the AP-1 transcription factor network pathway was over-represented by genes affected by trans eQTLs. Lastly, pathways over-represented by DEGs included the estrogen and GnRH signaling pathways (Figure 4A) and biological processes related to immune response (Figure 4B).

## 4. Discussion

We identified 3157 genes expressed in the uterine luminal epithelial cells affected by at least one eQTL. While these variants were mainly found in the Chr 18, 19, 23, and 25, it is important to highlight that they were spread across all autosomes. The eQTLs can affect the gene expression by different mechanisms, which include changing the sequence of regulatory elements and the affinity of the TF- and miRNA-binding sites [39]. Here, we found SNPs mainly harbored in downstream or 3′ UTR regions. Polymorphisms in the 3′ UTR may affect the post-transcriptional and translational processes due to their role in mRNA stability, translation, and miRNA binding [40]. Our analysis identified 31.1% of missense variants. Despite the impact of these variants resulting in a new amino acid, it has been shown that they have a low genetic importance to complex traits, likely due to purifying selection [41]. However, we must consider that, while these may not be the causal SNPs, they could be in LD with the causative one [9].

Our integration analysis using MatrixEQTL identified 4676 cis and 14,680 trans eQTLs. These findings support a previous report that trans eQTLs are more common in the genome [42]. The identified eQTLs were associated with the expression of TF coding genes and DEGs between pregnant and nonpregnant (P and NP) cows. Proteins encoded by TFs act by recognizing and binding specific sites of their target genes to either increase or repress gene expression [43]. The eQTLs located in the TF binding sites are associated not only with the TF itself, but can indirectly affect histone modification, the DNA structure, and mRNA expression [39]. Among the genes, 71 and 193 TFs were affected by cis and trans eQTLs, respectively. Although most of the targeted genes were not coding TFs, changes in one gene can affect the expression of others, as they share similar functional pathways [44].

Additionally, noncoding RNAs (ncRNAs), including lncRNAs and miRNAs, were targeted by both cis and trans eQTLs in our study. Noncoding RNAs have been associated with endometrial function and uterine receptivity by fine-tuning the expression of target genes in response to hormonal changes [45]. Fonseca et al. [46] identified 17 and 2 lncRNAs among co-expressed gene modules from endometrium associated with high- and subfertile cows, respectively.

A coordinated expression of uterine genes is critical to establish endometrial receptivity and embryo survival [8]. Genes involved with hormone signaling, nutrient transport, and metabolism were identified in our analyses. Pathways over-represented by cis-targeted genes included the tryptophan metabolism, phenylalanine, and tyrosine catabolism. Endometrial epithelial secretions of nutrients during early pregnancy are critical to support embryo development [3,47]. França et al. [48] showed that the amino acid profile of uterine flushing differs between receptive and nonreceptive cows. Likewise, Forde et al. [49] reported increasing tryptophan concentrations on day 16 of pregnancy.

We identified two (*ZNF470* and *SNAPC4*) and three (*AR*, *KLF7*, and *PPARG*) TFs affected by cis and trans eQTLs, respectively, which were previously reported as DEGs [14] in the same cows used in the current study. As expected, these TFs were over-represented in pathways related to gene transcription. Among the TFs, *AR* was over-represented in the androgen receptor and FOXA1 transcription factor network pathways. This TF regulates gene expression by mediating the actions of androgens, which stimulate the proliferation of the uterine epithelium cells [50,51]. Zhang et al. [52] reported that AR protein abundance affects the expression of genes involved with endometrial receptivity and decidualization in rats. Previous studies have reported an interaction between *AR* and *FOXA1* in cell proliferation related to human cancer [53,54]. In the current study, *FOXA1* was also affected by one trans eQTL (novel SNP). *FOXA1* encodes a TF that changes the chromatin structure to bind additional TFs, including *AR* [53]. Also involved with uterine receptivity, we found the TFs *FOXO1* and *GATA2* over-represented in the androgen receptor activity pathway. In humans, *FOXO1* is critical for embryo implantation [55], while *GATA2* coordinates gene expression mediated by progesterone [56].

Processes involved with uterine receptivity and pregnancy establishment include coordinated changes in estrogen and progesterone (P4) production [57]. While circulating P4 concentrations during the first 20 days postestrus were not different in beef heifers classified as high-fertile, subfertile, or infertile [13], other studies have shown that P4 affected the endometrial transcriptome and uterine function [57,58]. Martins et al. [14] suggested increased oxidative phosphorylation, biosynthetic activity, and the proliferation of uterine epithelial cells in response to P4 modulation. Additionally, they reported that the P4 concentration was associated with the differential expression of the endometrial luminal epithelium. Silva et al. [15] reported that the uterine transcriptome of cows with contrasting concentrations of P4-affected genes underlying immune signaling, extracellular matrix remodeling, and inflammatory response at days 4, 7, and 14 postestrus, respectively. Similar pathways were reported by Mazzoni et al. [59] at days 6–8 of the estrus as differentially regulated between pregnant and nonpregnant Holstein cows. These authors suggested that differences in the endometrial transcriptome, rather than hormonal variations, would be the main factor underlying receptivity. Collectively, these findings highlight the importance of genes and pathways for pregnancy establishment.

From the DEG list reported by Martins et al. [14], we identified 75 unique genes affected by eQTLs (32 cis and 56 trans). Interestingly, Chr 18 and 19 were also over-represented by DEGs. These chromosomes harbored most of the eQTLs identified here. The assumption is that these gene clusters act on the same pathways, which supports the hypothesis of a cascade effect in gene expression due to gene variants. The functional over-representation analysis of DEGs highlighted the estrogen and gonadotropin-releasing hormone (GnRH) signaling pathways, which included eQTL-affected genes (*RARA* and *ITPR3*). These are key regulators of reproductive processes, acting in a coordinated feedback system to regulate fertility [60]. Genes acting on the estrogen and GnRH signaling pathways were upregulated in the endometrium of cows with high progesterone [61]. The *RARA* (retinoic acid receptor alpha) TF was over-represented in these pathways. Retinoic acid (RA) signaling is critical to uterine receptivity and decidualization [62]. Musavi et al. [63] reported the *RARA* TF as an upstream regulator of DEGs at the follicular stage in the bovine endometrium.

The maternal innate and adaptive immune systems are critical to creating a conducive environment for the developing embryo [64]. Here, immune-related biological processes affected by eQTLs included antigen processing and the presentation of peptide antigen via the MHC class I and immune response. The genes underlying these pathways were mainly from the MHC family (*BOLA*, *BOLA-DQA1*, *BOLA-DQA2*, and *BOLA-DQB*). Likewise, lymphocyte activation, leucocyte activation, and immune response were BPs over-represented by the DEGs. Martins et al. [14] reported a downregulation of immune-associated genes from the endometrial luminal epithelial cells of pregnant cows. The role of the immune system at early diestrus for the establishment of pregnancy remains unclear [14]. However, its activation and a balance between pro- and anti-inflammatory molecules are required during initial placentation [65].

On the other hand, a higher expression of the complement system and communication between innate and adaptive immune cell pathways were found by Musavi et al. [63] in the endometrium of cows during the implantation stage (day 18). During this period, increasing levels of INF-τ significantly changed the maternal immune response [14,66]. The INF-τ hormone has been suggested as a key regulator of maternal immune response by modulating the genes involved with uterine receptivity, implantation, and conceptus development [67]. These findings show that differences in the immune response are important for pregnancy establishment even before increases in the concentration of INF-τ [14,16,59]. It is important to note that samples in the current study were collected before ET [14], thus there was no circulating INF-τ. According to [68], there is an association between the increased postovulatory P4 concentration and the increased INF-τ production. Therefore, SNPs increasing the expression of key genes would potentially improve uterine receptivity.

Several eQTLs overlapped with QTLs previously associated with reproduction traits. QTLs harbored in Ch 25 were the most over-represented, including traits such as the interval from the first estrus to the last insemination and the interval to the first estrus after calving. Likewise, cis eQTLs in Chr 3 overlapped with the luteal activity QTL. Primiparous Holstein cows carrying a favorable haplotype for fertility showed earlier luteal activity compared to the ones with the unfavorable haplotype [69]. However, the authors highlighted that factors other than ovarian dynamics were associated with differences in fertility [69]. Altogether, these findings provide insights into the regulatory effects of eQTL-targeted genes and their association with QTL regions that are important for reproduction-related traits. While we reported several gene targets, our approach does not provide the molecular mechanism by which changes in gene expression due to gene variants influence pregnancy outcomes. Hence, future studies will require larger sample sizes to validate these findings. Furthermore, the role of eQTL-affected genes on endometrial function and pregnancy establishment warrants further investigation.

Despite these findings, our study has some limitations. The SNPs were exclusively identified from transcribed regions on the RNA-Seq data, restricting the number of variants detected throughout the genome. Although we successfully identified eQTLs, none were directly associated with pregnancy outcomes. Similarly, the identified eQTLs were not validated in a different dataset. The lack of association may be due to the number of SNPs tested and the limited sample size (n = 43) used in the current study. Additionally, we used pregnancy status as the trait for association. Pregnancy is a binary trait affected by genetic, endocrine, and physiological factors, which brings more complexity to untangle the impact of associated variants [9]. Moreover, our study was based on a single-time-point analysis, which does not capture the dynamic nature of gene expression. Therefore, the associations reported here should be validated in a different cohort with a large sample size and a higher SNP coverage. Conducting expression analysis of multiple time points could provide a more comprehensive understanding of the temporal changes in eQTL regulation. Confirming results in easily sampled tissues like blood would also offer an opportunity for routine analysis. These suggestions aim to guide further investigations and refine our understanding of the molecular mechanisms underlying gene regulation and their functional implications to pregnancy.

## 5. Conclusions

We conducted a comprehensive eQTL analysis to unveil the genetic regulation of the uterine luminal epithelial cells of beef cows. These eQTLs were distributed across different chromosomes and overlapped reproduction-related QTLs. Additionally, we identified transcription factors and noncoding RNAs regulating gene expression that were affected by eQTLs. Functional over-representation analysis highlighted the pathways related to the metabolism, immune response, and hormone signaling, emphasizing their importance in successful pregnancy establishment. While further research is needed to validate these findings and understand the precise mechanisms involved, our study provides insights into the genetic basis of reproductive processes in cattle.

## Figures and Tables

**Table 1:** Top-ten genes affected by cis and trans eQTLs identified from bovine uterine luminal epithelial cells.

Ensembl ID	Gene Name	Number of eQTLs		
		Cis	Trans	Total
*ENSBTAG00000052527*	*ENSBTAG00000052527*	37	178	215
*ENSBTAG00000053827*	*ENSBTAG00000053827*	93	113	206
*ENSBTAG00000007816*	*ENSBTAG00000007816*	3	195	198
*ENSBTAG00000027397*	*PPP1R3D*	1	192	193
*ENSBTAG00000048353*	*ENSBTAG00000048353*	30	151	181
*ENSBTAG00000048470*	*IFITM1*	33	143	176
*ENSBTAG00000027075*	*ENSBTAG00000027075*	2	171	173
*ENSBTAG00000038050*	*ZNF420*	35	128	163
*ENSBTAG00000025782*	*TNFSF8*	1	137	138
*ENSBTAG00000033449*	*ENSBTAG00000033449*	1	128	129

**Table 2:** Enriched chromosomes by differentially expressed genes identified from bovine uterine luminal epithelial cells.

Enriched Chromosomes ^a^	Number of Genes	Genes ^b^
18	21	***ARHGEF1***, ***CLASRP***, *ENSBTAG00000023367*, *BICRA*, *LMTK3*, *PRR12*, *NAPSA*, *ENSBATG00000049577*, ***CD37***, *ENSBATG00000045880*, ***ENSBTAG00000051367***, *ENSBTAG00000053237*, *VSTM1*, *ZNF581*, ***KMT5C***, *PTPRH*, ***PPP6R1***, ***TMEM86B***, *EPS8L1*, ***SMIM17***, ***ZNF470***
19	24	*SCARF1*, ***P2RX5***,***PITPNM3***, *VMO1*, *CAMTA2*, *PLD2*, *TMEM95*, ***DLG4***, *KCTD11*, *NLGN2*, *SOX15*, *TMEM88*, *KDM6B*, ***PIK3R5***, *GAS7*, *RARA*, *ENSBTAG00000024839*, *STAT5A*, *CCR10*, *CNTNAP1*, ***TMEM106A***, *HIGD1B*, *FMNL1*, *ENSBTAG00000055302*
22	5	*SYN2*, ***PPARG***, ***CHST13***, *MGLL*, *SLC41A3*
25	8	*LAT*, *CD19*, *GDPD3*, *DOC2A*, *ENSBTAG00000046752*, *TMEM265*, ***FBXL19***, ***SEPTIN14***

^a^ FDR < 0.05; ^b^ Genes affected by cis or trans eQTLs are in orange or red, respectively. Black bolded genes are targeted by both cis and trans eQTLs. Differentially expressed genes were retrieved from Martins et al. [14].

## Data Availability

All relevant data are within the paper and its Supplementary Information files. All sequencing data are publicly available on Gene Expression Omnibus (GSE171577).

## Supplementary Materials

The following supporting information can be downloaded at: https://www.mdpi.com/article/10.3390/biom14020150/s1, Supplementary Table S1: Summary and mapping statistics of RNA-Sequencing data from uterine epithelium cells in beef cows; Supplementary Table S2: The VEP annotation of all variants and annotations of variants which change the protein coding sequence from bovine uterine luminal epithelium cells; Supplementary Table S3: Gene annotation and chromosome distribution of cis eQTLs from uterine epithelium cells in beef cows; Supplementary Table S4: Gene annotation and chromosome distribution of trans eQTLs from uterine epithelium cells in beef cows; Supplementary Table S5: Genes from bovine uterine luminal epithelium cells regulated by both cis and trans eQTLs; Supplementary Table S6: List of differentially expressed genes reported by Martins et al. [14] and over-represented biological processes; Supplementary Table S7: KEGG pathways and biological processes over-representation analyses based on cis eQTL-targeted genes and transcription factors from uterine epithelium cells in beef cows; Supplementary Table S8: KEGG pathways and biological processes over-representation analyses based on trans eQTL-targeted genes and transcription factors from uterine epithelium cells in beef cows; Supplementary Table S9: KEGG pathways and biological processes over-representation analyses based on genes targeted by both cis and trans eQTLs from uterine epithelium cells in beef cows.
